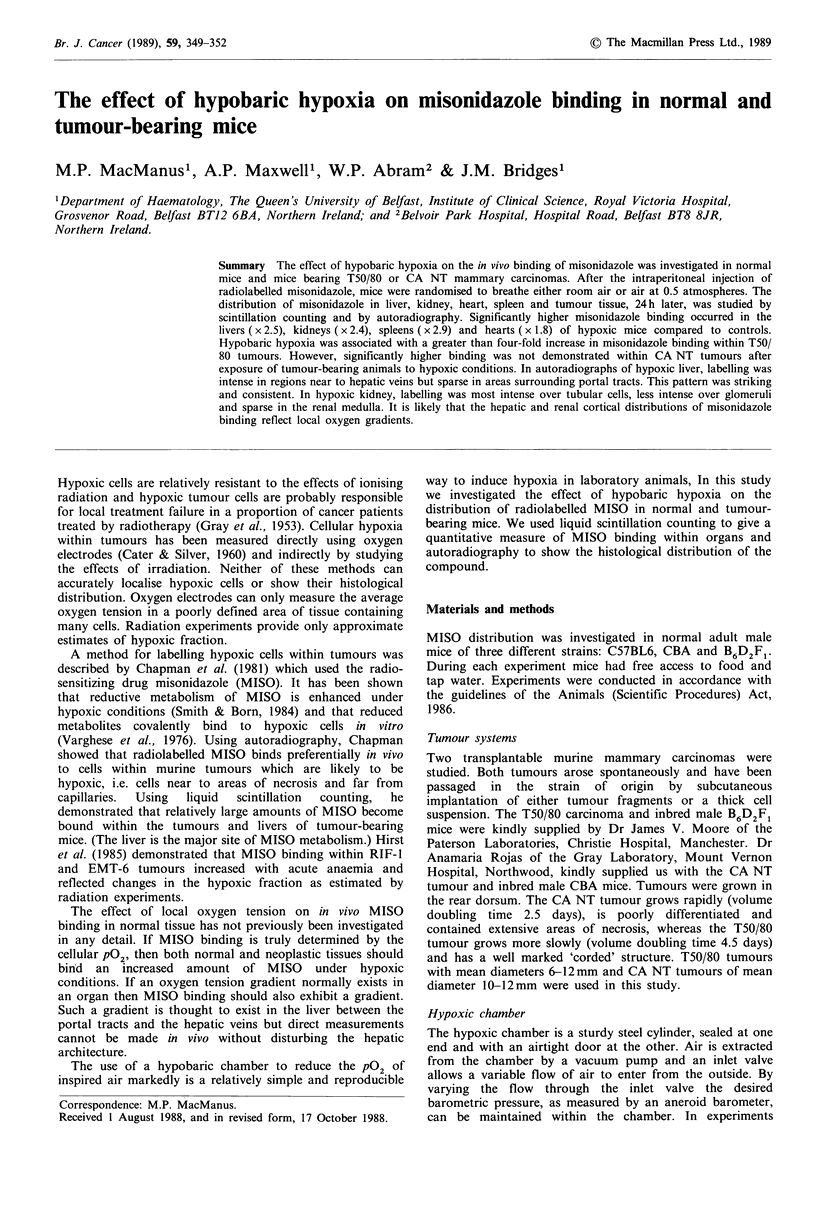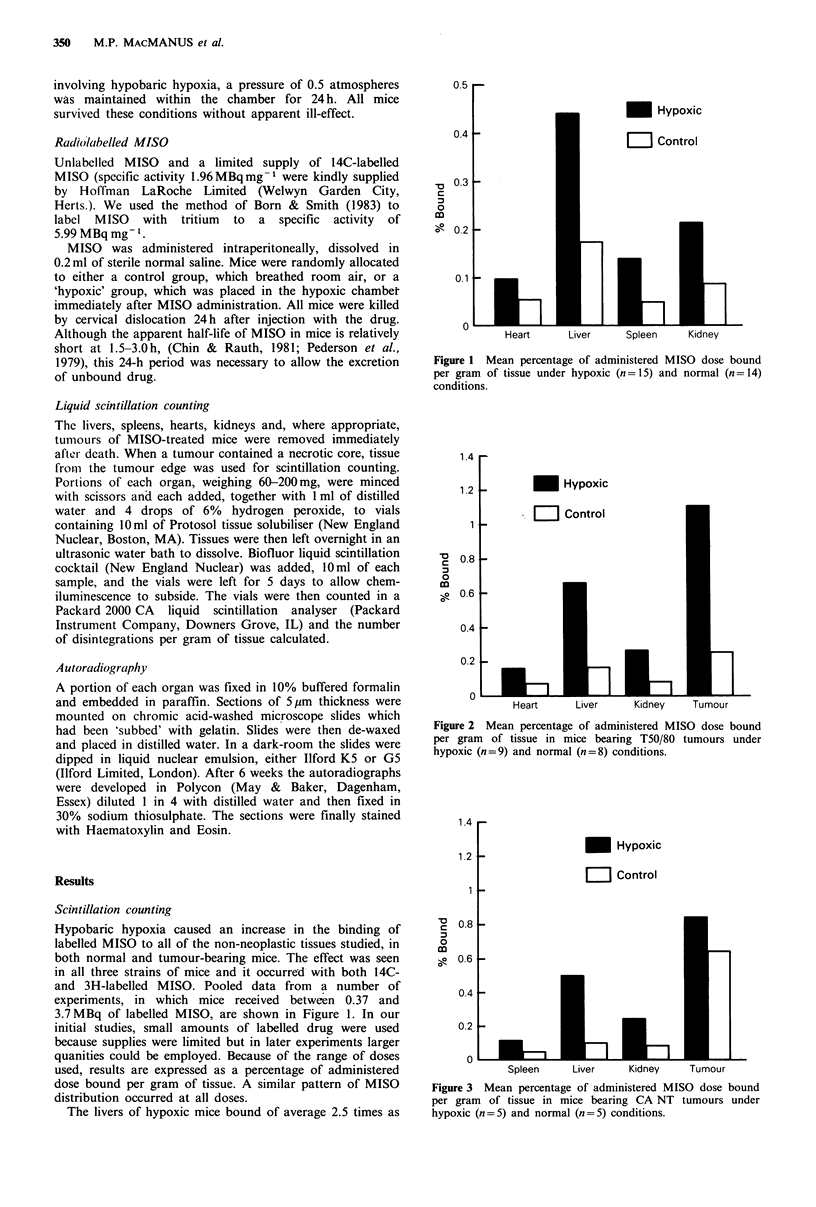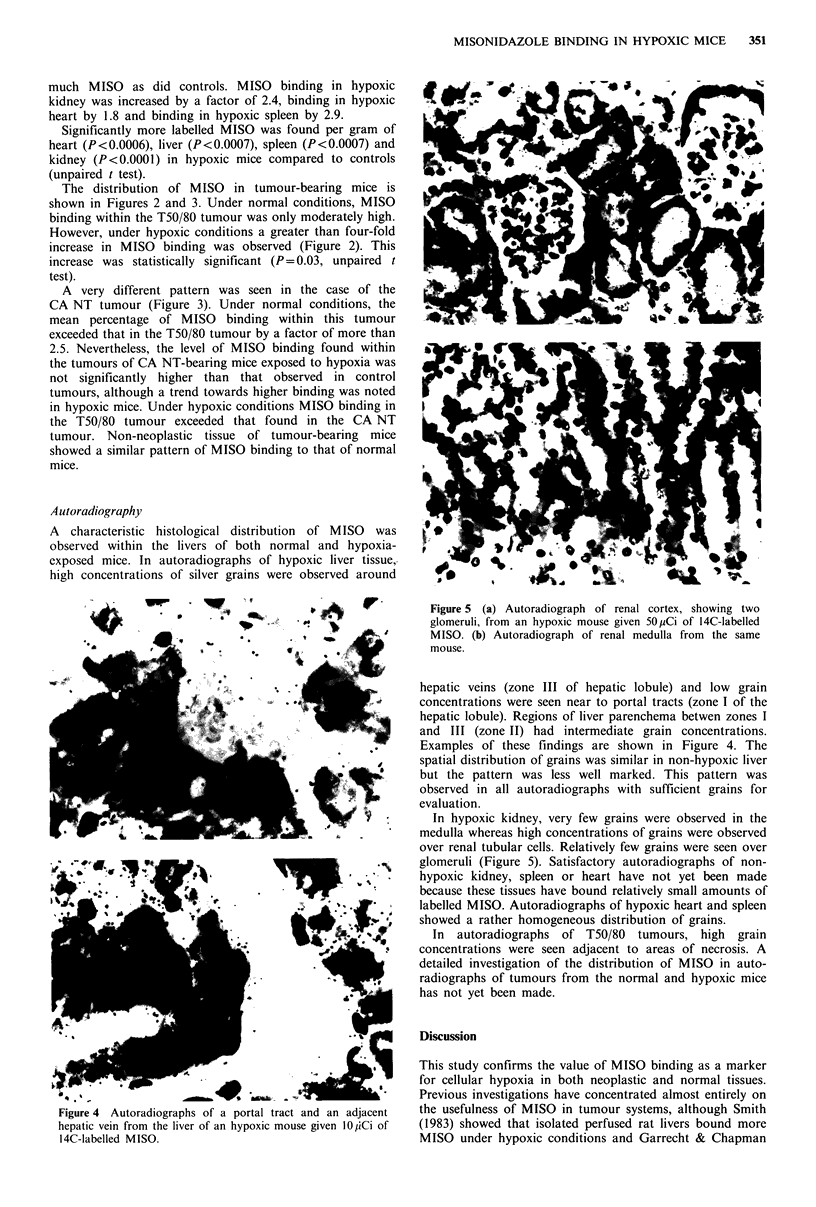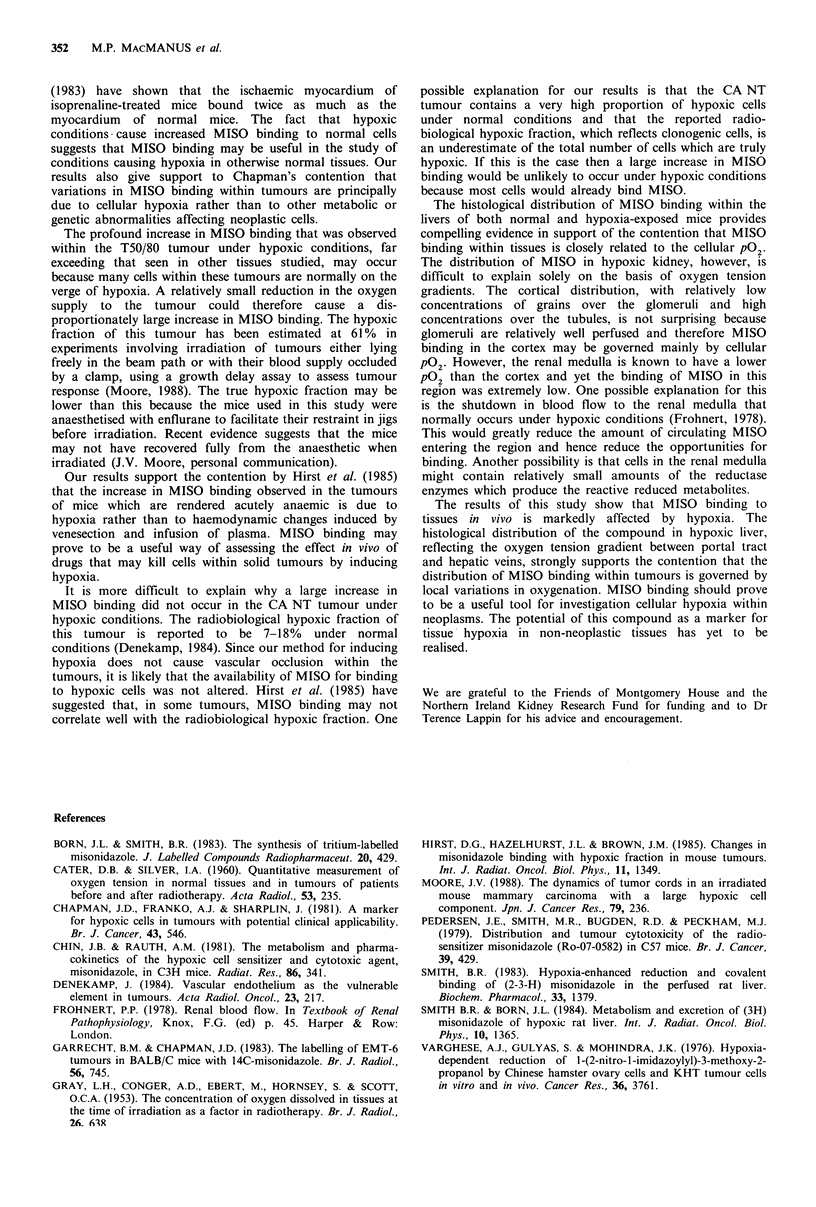# The effect of hypobaric hypoxia on misonidazole binding in normal and tumour-bearing mice.

**DOI:** 10.1038/bjc.1989.69

**Published:** 1989-03

**Authors:** M. P. MacManus, A. P. Maxwell, W. P. Abram, J. M. Bridges

**Affiliations:** Department of Haematology, Queen's University of Belfast, Royal Victoria Hospital, Northern Ireland.

## Abstract

**Images:**


*Br. J. Cancer* (1989), **59**, 349–352 © The Macmillan Press Ltd., 1989

### The effect of hypobaric hypoxia on misonidazole binding in normal and tumour-bearing mice

M.P. MacManus[1], A.P. Maxwell[1], W.P. Abram[2] & J.M. Bridges[1]

[1]*Department of Haematology, The Queen's University of Belfast, Institute of Clinical Science, Royal Victoria Hospital, Grosvenor Road, Belfast BT12 6BA, Northern Ireland; and* [2]*Belvoir Park Hospital, Hospital Road, Belfast BT8 8JR, Northern Ireland.*

**Summary** The effect of hypobaric hypoxia on the *in vivo* binding of misonidazole was investigated in normal mice and mice bearing T50/80 or CA NT mammary carcinomas. After the intraperitoneal injection of radiolabelled misonidazole, mice were randomised to breathe either room air or air at 0.5 atmospheres. The distribution of misonidazole in liver, kidney, heart, spleen and tumour tissue, 24 h later, was studied by scintillation counting and by autoradiography. Significantly higher misonidazole binding occurred in the livers ($\times 2.5$), kidneys ($\times 2.4$), spleens ($\times 2.9$) and hearts ($\times 1.8$) of hypoxic mice compared to controls. Hypobaric hypoxia was associated with a greater than four-fold increase in misonidazole binding within T50/80 tumours. However, significantly higher binding was not demonstrated within CA NT tumours after exposure of tumour-bearing animals to hypoxic conditions. In autoradiographs of hypoxic liver, labelling was intense in regions near to hepatic veins but sparse in areas surrounding portal tracts. This pattern was striking and consistent. In hypoxic kidney, labelling was most intense over tubular cells, less intense over glomeruli and sparse in the renal medulla. It is likely that the hepatic and renal cortical distributions of misonidazole binding reflect local oxygen gradients.

Hypoxic cells are relatively resistant to the effects of ionising radiation and hypoxic tumour cells are probably responsible for local treatment failure in a proportion of cancer patients treated by radiotherapy (Gray *et al.*, 1953). Cellular hypoxia within tumours has been measured directly using oxygen electrodes (Cater & Silver, 1960) and indirectly by studying the effects of irradiation. Neither of these methods can accurately localise hypoxic cells or show their histological distribution. Oxygen electrodes can only measure the average oxygen tension in a poorly defined area of tissue containing many cells. Radiation experiments provide only approximate estimates of hypoxic fraction.

A method for labelling hypoxic cells within tumours was described by Chapman *et al.* (1981) which used the radiosensitizing drug misonidazole (MISO). It has been shown that reductive metabolism of MISO is enhanced under hypoxic conditions (Smith & Born, 1984) and that reduced metabolites covalently bind to hypoxic cells *in vitro* (Varghese *et al.*, 1976). Using autoradiography, Chapman showed that radiolabelled MISO binds preferentially *in vivo* to cells within murine tumours which are likely to be hypoxic, i.e. cells near to areas of necrosis and far from capillaries. Using liquid scintillation counting, he demonstrated that relatively large amounts of MISO become bound within the tumours and livers of tumour-bearing mice. (The liver is the major site of MISO metabolism.) Hirst *et al.* (1985) demonstrated that MISO binding within RIF-1 and EMT-6 tumours increased with acute anaemia and reflected changes in the hypoxic fraction as estimated by radiation experiments.

The effect of local oxygen tension on *in vivo* MISO binding in normal tissue has not previously been investigated in any detail. If MISO binding is truly determined by the cellular $pO_2$, then both normal and neoplastic tissues should bind an increased amount of MISO under hypoxic conditions. If an oxygen tension gradient normally exists in an organ then MISO binding should also exhibit a gradient. Such a gradient is thought to exist in the liver between the portal tracts and the hepatic veins but direct measurements cannot be made *in vivo* without disturbing the hepatic architecture.

The use of a hypobaric chamber to reduce the $pO_2$ of inspired air markedly is a relatively simple and reproducible way to induce hypoxia in laboratory animals, In this study we investigated the effect of hypobaric hypoxia on the distribution of radiolabelled MISO in normal and tumour-bearing mice. We used liquid scintillation counting to give a quantitative measure of MISO binding within organs and autoradiography to show the histological distribution of the compound.

Correspondence: M.P. MacManus.
Received 1 August 1988, and in revised form, 17 October 1988.

### Materials and methods

MISO distribution was investigated in normal adult male mice of three different strains: C57BL6, CBA and $B_6D_2F_1$. During each experiment mice had free access to food and tap water. Experiments were conducted in accordance with the guidelines of the Animals (Scientific Procedures) Act, 1986.

### *Tumour systems*

Two transplantable murine mammary carcinomas were studied. Both tumours arose spontaneously and have been passaged in the strain of origin by subcutaneous implantation of either tumour fragments or a thick cell suspension. The T50/80 carcinoma and inbred male $B_6D_2F_1$ mice were kindly supplied by Dr James V. Moore of the Paterson Laboratories, Christie Hospital, Manchester. Dr Anamaria Rojas of the Gray Laboratory, Mount Vernon Hospital, Northwood, kindly supplied us with the CA NT tumour and inbred male CBA mice. Tumours were grown in the rear dorsum. The CA NT tumour grows rapidly (volume doubling time 2.5 days), is poorly differentiated and contained extensive areas of necrosis, whereas the T50/80 tumour grows more slowly (volume doubling time 4.5 days) and has a well marked 'corded' structure. T50/80 tumours with mean diameters 6–12 mm and CA NT tumours of mean diameter 10–12 mm were used in this study.

### *Hypoxic chamber*

The hypoxic chamber is a sturdy steel cylinder, sealed at one end and with an airtight door at the other. Air is extracted from the chamber by a vacuum pump and an inlet valve allows a variable flow of air to enter from the outside. By varying the flow through the inlet valve the desired barometric pressure, as measured by an aneroid barometer, can be maintained within the chamber. In experiments involving hypobaric hypoxia, a pressure of 0.5 atmospheres was maintained within the chamber for 24 h. All mice survived these conditions without apparent ill-effect.

*Radiolabelled MISO*

Unlabelled MISO and a limited supply of 14C-labelled MISO (specific activity 1.96 MBq $mg^{-1}$ were kindly supplied by Hoffman LaRoche Limited (Welwyn Garden City, Herts.). We used the method of Born & Smith (1983) to label MISO with tritium to a specific activity of 5.99 MBq $mg^{-1}$.

MISO was administered intraperitoneally, dissolved in 0.2 ml of sterile normal saline. Mice were randomly allocated to either a control group, which breathed room air, or a 'hypoxic' group, which was placed in the hypoxic chamber immediately after MISO administration. All mice were killed by cervical dislocation 24 h after injection with the drug. Although the apparent half-life of MISO in mice is relatively short at 1.5–3.0 h, (Chin & Rauth, 1981; Pederson *et al.*, 1979), this 24-h period was necessary to allow the excretion of unbound drug.

*Liquid scintillation counting*

The livers, spleens, hearts, kidneys and, where appropriate, tumours of MISO-treated mice were removed immediately after death. When a tumour contained a necrotic core, tissue from the tumour edge was used for scintillation counting. Portions of each organ, weighing 60–200 mg, were minced with scissors and each added, together with 1 ml of distilled water and 4 drops of 6% hydrogen peroxide, to vials containing 10 ml of Protosol tissue solubiliser (New England Nuclear, Boston, MA). Tissues were then left overnight in an ultrasonic water bath to dissolve. Biofluor liquid scintillation cocktail (New England Nuclear) was added, 10 ml of each sample, and the vials were left for 5 days to allow chemiluminescence to subside. The vials were then counted in a Packard 2000 CA liquid scintillation analyser (Packard Instrument Company, Downers Grove, IL) and the number of disintegrations per gram of tissue calculated.

*Autoradiography*

A portion of each organ was fixed in 10% buffered formalin and embedded in paraffin. Sections of 5 $\mu$m thickness were mounted on chromic acid-washed microscope slides which had been 'subbed' with gelatin. Slides were then de-waxed and placed in distilled water. In a dark-room the slides were dipped in liquid nuclear emulsion, either Ilford K5 or G5 (Ilford Limited, London). After 6 weeks the autoradiographs were developed in Polycon (May & Baker, Dagenham, Essex) diluted 1 in 4 with distilled water and then fixed in 30% sodium thiosulphate. The sections were finally stained with Haematoxylin and Eosin.

### Results

*Scintillation counting*

Hypobaric hypoxia caused an increase in the binding of labelled MISO to all of the non-neoplastic tissues studied, in both normal and tumour-bearing mice. The effect was seen in all three strains of mice and it occurred with both 14C- and 3H-labelled MISO. Pooled data from a number of experiments, in which mice received between 0.37 and 3.7 MBq of labelled MISO, are shown in Figure 1. In our initial studies, small amounts of labelled drug were used because supplies were limited but in later experiments larger quanities could be employed. Because of the range of doses used, results are expressed as a percentage of administered dose bound per gram of tissue. A similar pattern of MISO distribution occurred at all doses.

The livers of hypoxic mice bound of average 2.5 times as

**Figure 1** Mean percentage of administered MISO dose bound per gram of tissue under hypoxic ($n=15$) and normal ($n=14$) conditions.

**Figure 2** Mean percentage of administered MISO dose bound per gram of tissue in mice bearing T50/80 tumours under hypoxic ($n=9$) and normal ($n=8$) conditions.

**Figure 3** Mean percentage of administered MISO dose bound per gram of tissue in mice bearing CA NT tumours under hypoxic ($n=5$) and normal ($n=5$) conditions.

much MISO as did controls. MISO binding in hypoxic kidney was increased by a factor of 2.4, binding in hypoxic heart by 1.8 and binding in hypoxic spleen by 2.9.

Significantly more labelled MISO was found per gram of heart ($P<0.0006$), liver ($P<0.0007$), spleen ($P<0.0007$) and kidney ($P<0.0001$) in hypoxic mice compared to controls (unpaired *t* test).

The distribution of MISO in tumour-bearing mice is shown in Figures 2 and 3. Under normal conditions, MISO binding within the T50/80 tumour was only moderately high. However, under hypoxic conditions a greater than four-fold increase in MISO binding was observed (Figure 2). This increase was statistically significant ($P=0.03$, unpaired *t* test).

A very different pattern was seen in the case of the CA NT tumour (Figure 3). Under normal conditions, the mean percentage of MISO binding within this tumour exceeded that in the T50/80 tumour by a factor of more than 2.5. Nevertheless, the level of MISO binding found within the tumours of CA NT-bearing mice exposed to hypoxia was not significantly higher than that observed in control tumours, although a trend towards higher binding was noted in hypoxic mice. Under hypoxic conditions MISO binding in the T50/80 tumour exceeded that found in the CA NT tumour. Non-neoplastic tissue of tumour-bearing mice showed a similar pattern of MISO binding to that of normal mice.

### *Autoradiography*

A characteristic histological distribution of MISO was observed within the livers of both normal and hypoxia-exposed mice. In autoradiographs of hypoxic liver tissue, high concentrations of silver grains were observed around

**Figure 4** Autoradiographs of a portal tract and an adjacent hepatic vein from the liver of an hypoxic mouse given 10 μCi of 14C-labelled MISO.

**Figure 5** (**a**) Autoradiograph of renal cortex, showing two glomeruli, from an hypoxic mouse given 50 μCi of 14C-labelled MISO. (**b**) Autoradiograph of renal medulla from the same mouse.

hepatic veins (zone III of hepatic lobule) and low grain concentrations were seen near to portal tracts (zone I of the hepatic lobule). Regions of liver parenchema betwen zones I and III (zone II) had intermediate grain concentrations. Examples of these findings are shown in Figure 4. The spatial distribution of grains was similar in non-hypoxic liver but the pattern was less well marked. This pattern was observed in all autoradiographs with sufficient grains for evaluation.

In hypoxic kidney, very few grains were observed in the medulla whereas high concentrations of grains were observed over renal tubular cells. Relatively few grains were seen over glomeruli (Figure 5). Satisfactory autoradiographs of non-hypoxic kidney, spleen or heart have not yet been made because these tissues have bound relatively small amounts of labelled MISO. Autoradiographs of hypoxic heart and spleen showed a rather homogeneous distribution of grains.

In autoradiographs of T50/80 tumours, high grain concentrations were seen adjacent to areas of necrosis. A detailed investigation of the distribution of MISO in autoradiographs of tumours from the normal and hypoxic mice has not yet been made.

### Discussion

This study confirms the value of MISO binding as a marker for cellular hypoxia in both neoplastic and normal tissues. Previous investigations have concentrated almost entirely on the usefulness of MISO in tumour systems, although Smith (1983) showed that isolated perfused rat livers bound more MISO under hypoxic conditions and Garrecht & Chapman

(1983) have shown that the ischaemic myocardium of isoprenaline-treated mice bound twice as much as the myocardium of normal mice. The fact that hypoxic conditions cause increased MISO binding to normal cells suggests that MISO binding may be useful in the study of conditions causing hypoxia in otherwise normal tissues. Our results also give support to Chapman's contention that variations in MISO binding within tumours are principally due to cellular hypoxia rather than to other metabolic or genetic abnormalities affecting neoplastic cells.

The profound increase in MISO binding that was observed within the T50/80 tumour under hypoxic conditions, far exceeding that seen in other tissues studied, may occur because many cells within these tumours are normally on the verge of hypoxia. A relatively small reduction in the oxygen supply to the tumour could therefore cause a disproportionately large increase in MISO binding. The hypoxic fraction of this tumour has been estimated at 61% in experiments involving irradiation of tumours either lying freely in the beam path or with their blood supply occluded by a clamp, using a growth delay assay to assess tumour response (Moore, 1988). The true hypoxic fraction may be lower than this because the mice used in this study were anaesthetised with enflurane to facilitate their restraint in jigs before irradiation. Recent evidence suggests that the mice may not have recovered fully from the anaesthetic when irradiated (J.V. Moore, personal communication).

Our results support the contention by Hirst *et al.* (1985) that the increase in MISO binding observed in the tumours of mice which are rendered acutely anaemic is due to hypoxia rather than to haemodynamic changes induced by venesection and infusion of plasma. MISO binding may prove to be a useful way of assessing the effect *in vivo* of drugs that may kill cells within solid tumours by inducing hypoxia.

It is more difficult to explain why a large increase in MISO binding did not occur in the CA NT tumour under hypoxic conditions. The radiobiological hypoxic fraction of this tumour is reported to be 7–18% under normal conditions (Denekamp, 1984). Since our method for inducing hypoxia does not cause vascular occlusion within the tumours, it is likely that the availability of MISO for binding to hypoxic cells was not altered. Hirst *et al.* (1985) have suggested that, in some tumours, MISO binding may not correlate well with the radiobiological hypoxic fraction. One possible explanation for our results is that the CA NT tumour contains a very high proportion of hypoxic cells under normal conditions and that the reported radiobiological hypoxic fraction, which reflects clonogenic cells, is an underestimate of the total number of cells which are truly hypoxic. If this is the case then a large increase in MISO binding would be unlikely to occur under hypoxic conditions because most cells would already bind MISO.

The histological distribution of MISO binding within the livers of both normal and hypoxia-exposed mice provides compelling evidence in support of the contention that MISO binding within tissues is closely related to the cellular $pO_2$. The distribution of MISO in hypoxic kidney, however, is difficult to explain solely on the basis of oxygen tension gradients. The cortical distribution, with relatively low concentrations of grains over the glomeruli and high concentrations over the tubules, is not surprising because glomeruli are relatively well perfused and therefore MISO binding in the cortex may be governed mainly by cellular $pO_2$. However, the renal medulla is known to have a lower $pO_2$ than the cortex and yet the binding of MISO in this region was extremely low. One possible explanation for this is the shutdown in blood flow to the renal medulla that normally occurs under hypoxic conditions (Frohnert, 1978). This would greatly reduce the amount of circulating MISO entering the region and hence reduce the opportunities for binding. Another possibility is that cells in the renal medulla might contain relatively small amounts of the reductase enzymes which produce the reactive reduced metabolites.

The results of this study show that MISO binding to tissues *in vivo* is markedly affected by hypoxia. The histological distribution of the compound in hypoxic liver, reflecting the oxygen tension gradient between portal tract and hepatic veins, strongly supports the contention that the distribution of MISO binding within tumours is governed by local variations in oxygenation. MISO binding should prove to be a useful tool for investigation cellular hypoxia within neoplasms. The potential of this compound as a marker for tissue hypoxia in non-neoplastic tissues has yet to be realised.

We are grateful to the Friends of Montgomery House and the Northern Ireland Kidney Research Fund for funding and to Dr Terence Lappin for his advice and encouragement.

### References

BORN, J.L. & SMITH, B.R. (1983). The synthesis of tritium-labelled misonidazole. *J. Labelled Compounds Radiopharmaceut.* **20**, 429.

CATER, D.B. & SILVER, I.A. (1960). Quantitative measurement of oxygen tension in normal tissues and in tumours of patients before and after radiotherapy. *Acta Radiol.*, **53**, 235.

CHAPMAN, J.D., FRANKO, A.J. & SHARPLIN, J. (1981). A marker for hypoxic cells in tumours with potential clinical applicability. *Br. J. Cancer*, **43**, 546.

CHIN, J.B. & RAUTH, A.M. (1981). The metabolism and pharmacokinetics of the hypoxic cell sensitizer and cytotoxic agent, misonidazole, in C3H mice. *Radiat. Res.*, **86**, 341.

DENEKAMP, J. (1984). Vascular endothelium as the vulnerable element in tumours. *Acta Radiol. Oncol.*, **23**, 217.

FROHNERT, P.P. (1978). Renal blood flow. In *Textbook of Renal Pathophysiology*, Knox, F.G. (ed) p. 45. Harper & Row: London.

GARRECHT, B.M. & CHAPMAN, J.D. (1983). The labelling of EMT-6 tumours in BALB/C mice with 14C-misonidazole. *Br. J. Radiol.*, **56**, 745.

GRAY, L.H., CONGER, A.D., EBERT, M., HORNSEY, S. & SCOTT, O.C.A. (1953). The concentration of oxygen dissolved in tissues at the time of irradiation as a factor in radiotherapy. *Br. J. Radiol.*, **26**, 638.

HIRST, D.G., HAZELHURST, J.L. & BROWN, J.M. (1985). Changes in misonidazole binding with hypoxic fraction in mouse tumours. *Int. J. Radiat. Oncol. Biol. Phys.*, **11**, 1349.

MOORE, J.V. (1988). The dynamics of tumor cords in an irradiated mouse mammary carcinoma with a large hypoxic cell component. *Jpn. J. Cancer Res.*, **79**, 236.

PEDERSEN, J.E., SMITH, M.R., BUGDEN, R.D. & PECKHAM, M.J. (1979). Distribution and tumour cytotoxicity of the radiosensitizer misonidazole (Ro-07-0582) in C57 mice. *Br. J. Cancer*, **39**, 429.

SMITH, B.R. (1983). Hypoxia-enhanced reduction and covalent binding of (2-3-H) misonidazole in the perfused rat liver. *Biochem. Pharmacol.*, **33**, 1379.

SMITH B.R. & BORN, J.L. (1984). Metabolism and excretion of (3H) misonidazole of hypoxic rat liver. *Int. J. Radiat. Oncol. Biol. Phys.*, **10**, 1365.

VARGHESE, A.J., GULYAS, S. & MOHINDRA, J.K. (1976). Hypoxia-dependent reduction of 1-(2-nitro-1-imidazoylyl)-3-methoxy-2-propanol by Chinese hamster ovary cells and KHT tumour cells *in vitro* and *in vivo*. *Cancer Res.*, **36**, 3761.